# The impact of childhood temperament on the development of borderline personality disorder symptoms over the course of adolescence

**DOI:** 10.1186/2051-6673-1-18

**Published:** 2014-12-09

**Authors:** Stephanie D Stepp, Kate Keenan, Alison E Hipwell, Robert F Krueger

**Affiliations:** Department of Psychiatry, University of Pittsburgh School of Medicine, 3811 O’Hara St, Pittsburgh, PA USA; Department of Psychiatry and Behavioral Neuroscience, University of Chicago, 5841 S Maryland Ave, Chicago, IL USA; Department of Psychology, University of Minnesota, 75 E. River Rd, Minneapolis, MN USA

**Keywords:** Childhood temperament, Borderline personality disorder, Development, Girls

## Abstract

**Background:**

The purpose of this study was to characterize the development of BPD symptoms across adolescence by evaluating the fit of several latent variable growth models to annual assessments of symptoms obtained from girls when they were ages 14 through 19 years. After determining the best fitting model, we examined prospective associations between the temperament dimensions of emotionality, activity, low sociability, and shyness and BPD symptom development.

**Methods:**

We utilized longitudinal data from the Pittsburgh Girls Study; one of the few large-scale, prospective studies of girls (N = 2,450) in the United States. Parent- and teacher-reports of girls’ temperament were collected at Wave 1, when girls were ages 5–8 years. Child-reports of BPD symptoms were collected annually beginning at age 14 through 19 years.

**Results:**

We found that a free curve slope intercept model provided the best model fit, with the course of BPD symptoms characterized by a large component of inter-individual stability and a smaller component representing within-individual changes across adolescence. Symptoms appeared to peak by age 15, decline through age 18, and remain steady between ages 18 and 19 years. Both parent- and teacher-reports of temperament emotionality, activity, low sociability, and shyness predicted the developmental course of symptoms.

**Conclusions:**

BPD symptoms in adolescence reflect trait-like differences between youth with less within-person variability across time. Childhood temperament dimensions of emotionality, activity, low sociability, and shyness predict adolescent BPD symptom development. Parent- and teacher-informants provide unique information about the course of BPD symptoms, underscoring the utility of collecting child assessments using multiple informants.

## Background

Borderline personality disorder (BPD) emerges during adolescence or young adulthood and is characterized by multiple debilitating symptoms, including emotional dysregulation, tumultuous interpersonal relationships, and impulsive behaviors; all of which can interfere with occupational, academic, and social functioning [1–4]. Moreover, an estimated 8-10% of individuals with BPD will die by suicide, a rate 50 times greater than in the general population [5]. These devastating consequences speak to the urgent need to identify factors in early childhood that presage the onset and course of BPD symptomatology in adolescence. Successful identification of earlier risk factors would provide specific targets for early intervention aimed at deflecting the course of personality development away from BPD outcomes. For example, many theorists have suggested that BPD has early roots in childhood temperament [6–10]. In the current research, we sought to model how childhood temperament predicts the course of BPD symptoms throughout adolescence.

Little research exists about the development of BPD symptoms prior to adulthood and about how childhood temperament might predict parameters of developmental course. In particular, few studies have assessed BPD symptoms in youth across the multiple waves of data collection needed to characterize the pattern of adolescent growth. Findings from community samples have demonstrated that BPD symptoms and features peak during mid-adolescence and decline during late adolescence and young adulthood [11–14]. This pattern of within-individual change mirrors the normative decline in negative affectivity and disinhibition that occurs during the transition from adolescence to young adulthood [15, 16]. Some research has demonstrated that a significant portion of individuals may exhibit no change or may experience an increase in BPD-relevant personality traits during this developmental period [17, 18]. However, no study has compared a wide range of statistical models to describe the between- and within-individual differences in BPD symptoms over time. Instead, many studies have focused on estimating individual parameters within a single model (as opposed to contrasting the fit of different models, which may have different implications for understanding personality development [19, 20]. Critically evaluating such models allows the data to speak for themselves about the form of BPD symptom development. In the current study, we aim to fill this gap.

Given the developmental continuities between childhood temperament and personality, temperament is an obvious etiological feature to consider for the development of personality disorder symptoms in adolescence [21–23]. Childhood temperament reflects early individual differences in reactivity and self-regulation and can be conceptualized in terms of dimensions such as emotionality, activity, sociability, and shyness [24–26]. Childhood temperament and early emerging personality traits may be manifestations of the same basic, underlying dimensions [27–30]. Emotionality refers to how easily a child experiences and expresses negative emotions and is linked with the personality trait neuroticism. Activity level reflects children with high levels of energy who engage in a fast-paced lifestyle and at the extreme end may reflect personality traits such as disinhibition. Sociability reflects proclivities for social engagement and children high on this dimension might be described as gregarious and extroverted. Shyness refers to the tendency to be timid, withdrawn, and uncomfortable in social situations and can be linked to later introversion and inhibition. In contrast to children with low levels of sociability, children with high levels of shyness may desire social contact but find social engagement distressing, and as such, shyness also has associations with early emerging neurotic personality traits.

Consistent with DSM-5 Section III model of personality disorders, we can conceptualize personality disorders as extreme or maladaptive variants of normal personality traits, and, using this framework, BPD can be viewed as extremely high levels of neuroticism, low agreeableness, and low conscientiousness or high disinhibition [31–33]. There have been mixed results regarding the link between emotionality, impulsivity, and BPD [6, 8–10]. For instance, infant activity and emotionality, but not impulsivity, were related to BPD symptoms at age 28 [7]. Recent evidence also suggests links between adolescent BPD symptoms and adolescent temperament dimensions related to disinhibition and antagonism, but not emotionality [34, 35]. These inconsistencies may reflect methodological differences in the measurement of temperament, including the method of informant, as well as the timing of assessments across a wide span of developmental periods. The current study extends previous work on childhood temperament and BPD by including multiple informants of childhood temperament who do not overlap with the reporter for BPD symptoms. Additionally, this is the first study to examine how parent- and teacher-reports of temperament impact the developmental parameters of BPD symptoms over an extended developmental period from childhood through adolescence. Previous examinations have relied on contemporaneous assessments of temperament and BPD symptoms or have predicted BPD symptoms at one point in time. Based on previous findings between childhood temperament, adult personality traits, and BPD symptoms, we expected the temperament dimensions of high emotionality, high activity, low shyness, and low sociability to predict elevated and increasing BPD symptoms across ages 14–19 years.

We have previously examined the bi-directional associations between parenting and BPD symptom development in the context of child and parent characteristics using autoregressive latent trajectory models [14]. These models decomposed parenting and BPD symptoms into trait and state components and revealed moderate levels of trait-like stability in BPD symptoms across adolescence. Results from this investigation demonstrated that parental report of girls’ emotionality at age 11 was significantly associated with elevated BPD symptoms from ages 14 to 17 years. The current study extends this previous work in several critical ways. First, we compare the fit of several latent variable growth models to characterize the development of BPD symptoms across adolescence, extending the trajectory through age 19. Second, we investigate prospective associations between dimensions of temperament assessed at ages 5–8 years and BPD symptom development across adolescence when girls were 14–19 years old, which spans a much longer time frame than previously investigated. Third, we examine the utility of including both parent and teacher reports of child temperament in predicting BPD symptom development.

## Methods

### Sample description

The Pittsburgh Girls Study (PGS; *N* = 2,450) comprises an urban community sample of girls, ages 5–8 years at the first assessment, and their primary caregivers. To identify the study sample, low income neighborhoods were oversampled: neighborhoods that were characterized by at least 25% of families living at or below poverty level were fully enumerated and 50% of households in all other neighborhoods were randomly selected for enumeration as well [36, 37]. The current study used data collected at the first annual assessment (Wave 1) and across the most recent 7 assessment waves (Waves 7–13) to examine the associations between child temperament and trajectories of borderline personality disorder symptoms across adolescence. From the 2,450 participants, 2,282 (93.1%) provided data for at least one time point. Attrition analyses showed that girls with missing data across waves 6 through 13 were more likely to identify their race as European American (5% vs. 4%; *χ*^*2*^ = 20.23, df = 1, *p* < .001) and were less likely to report receipt of family public assistance at Wave 1 (6.6% vs. 2.5%, *χ*^*2*^ = 13.63, df = 1, *p* < .001).

African American girls made up slightly more than half of the sample (53%), and 41.2% were European American. Most of the remaining girls were identified by their caregivers as African American and another race; thus, 58.8% identified as minority race. More than one-third of the sample (38.9%) at Wave 1 received public assistance (e.g., Nutrition Program for Women, Infants, and Children; food stamps; Temporary Assistance for Needy Families). The overwhelming majority of caregivers were biological/birth parents (92.0%), and therefore, we refer to caregivers as parents. Most parents (57%) were cohabiting with a spouse or domestic partner; and 50% completed >12 years of education. Caregivers’ ages ranged from 21 to 83 years at Wave 1 (*M* = 37.76, *SD* = 8.57).

### Data collection procedures

Separate in-home interviews for both the girl and parent were conducted annually by trained interviewers using a laptop computer. All study procedures were approved by the University of Pittsburgh Institutional Review Board. Families were compensated for their participation.

### Measures

#### Temperament

Four temperament dimensions were measured using parent- and teacher-reports on the Emotionality, Activity, and Sociability (EAS) Temperament Survey [24] at Wave 1 (when girls were ages 5–8). The emotionality subscale consisted of five items (e.g., “She cries easily”), the activity subscale consisted of five items (e.g., “She is always on the go”), the shyness subscale was measured with two items (e.g., “She tends to be shy”) and the sociability subscale was measured with one item (“She is something of a loner”). The sociability item was scored so that higher scores reflected lower levels of sociability. All items were scored using a 5-point scale (1 = *a little* to 5 = *a lot*). In our study, the internal consistency coefficients for the parent-reported subscales were α = 0.82, α = 0.62, and α = 0.66 for emotionality, activity, and shyness, respectively. The internal consistency coefficients for the teacher-reported subscales were α = 0.88, α = 0.71, and α = 0.76 for emotionality, activity, and shyness, respectively. Internal consistencies for parent- and teacher-reports of sociability are not reported because, as noted previously, sociability was measured with one item.

#### BPD symptoms

Girls reported annually on their BPD symptoms beginning at age 14 years and through age 19 years using the screening questionnaire of the International Personality Disorders Examination (IPDE-BOR [38]), which is based on The Diagnostic and Statistical Manual of Mental Disorders, Fourth Edition (DSM-IV [39]) criteria for BPD. The IPDE-BOR consists of nine items (e.g., “I get into very intense relationships that don’t last”) scored either true or false. Adequate concurrent validity, and sensitivity and specificity of BPD symptom scores to clinicians’ diagnosis have been demonstrated for the IPDE-BOR in a sample of youth [40]. BPD symptoms demonstrated adequate internal consistency, as measured by alpha coefficient, at each age ranging from a high of 0.73 to a low of 0.69 at age 19. To examine the convergent validity of the IPDE-BOR with BPD symptom severity scores from a semi-structured clinical interview, we administered a semi-structured clinical interview, the Structured Interview for DSM-IV Personality Disorders [41], to a sub-sample of PGS participants (*n* = 65). In this sub-sample, the number of clinically significant symptoms reported on the SIDP-IV ranged from 0–7, with 20.1% reporting 0 symptoms, 66.9% reporting 1–4 symptoms, and 12.9% reporting 5 or more symptoms. We found a strong correlation between the IPDE-BOR and SID-IV symptom severity scores, (*r* = 0.71, *p* < .001), which supports the convergent validity of the IPDE-BOR as a measure of BPD symptoms.

### Data analytic plan

First, we calculated the zero-order correlations between demographic variables, child temperament domains, and adolescent BPD symptoms. Second, we estimated seven different latent variable growth models to determine the most appropriate form of BPD symptom development across adolescence. Specifically, we first examined a free curve slope intercept model (FCSI), which makes the fewest assumptions about the form or rate of growth [20, 42, 43]. Next, we estimated 6 additional growth models and compared their fit to the baseline FCSI model: (1) factor model with means (FM), (2) FM-Shift model, (3) MANOVA model, (4) MANOVA model without the compound symmetry assumption, (5) linear slope intercept model, and (6) quadratic slope intercept model.

Differences between the FCSI model and these more restrictive growth models are important to consider as they inform our understanding of BPD symptom development across adolescence. Specifically, the FM and FM-Shift models constrain growth to reflect within-individual change but not inter-individual, trait-like stability across time. The FM model estimates a factor mean but constrains all parameters associated with the latent intercept to zero. Given that the FM model assumes that manifest variables have meaningful zero scores, this could yield a relatively diminished model fit. The FM-Shift model relaxes this assumption by freely estimating the latent intercept variable mean, which “shifts” the curve by the interval-level scale represented in the data. If the FCSI model provides a better fit to the data (compared with the FM and/or FM-Shift models), then it suggests significant amounts of trait-like stability in BPD symptoms across ages 14 to 19.

In contrast to restricting growth to reflect only within-person change, the MANOVA models constrain the developmental trajectory to reflect only inter-individual or trait-like variability in the data. MANOVA assumes that measurement error is constant across time. By allowing measurement error to vary across assessment points, the assumption of compound symmetry is relaxed. If the MANOVA models fail to provide good fit to our data relative to the FCSI model, then we can infer that significant amounts of within-individual variability also exists in the development of BPD symptoms across adolescence.

Finally, we examined two growth models that assume a standardized rate of change across time. The linear slope intercept model constrains the latent slope variable to reflect a linear rate of growth (0, 1, 2, 3, 4, 5, and 6 for ages 14 through 19, respectively, for these data). Similarly, the quadratic slope intercept model adds a higher-order polynomial form of growth and constrains the quadratic latent variable by squaring the slope loadings (0, 1, 4, 9, 16, 25, and 36 for ages 14 through 19, respectively, for these data). The failure of linear and quadratic slope intercept models to provide good fit to our data would suggest that a constant, steady rate of change does not adequately describe the observed change in BPD symptoms.

All models except the quadratic slope intercept model were covariance nested within the FCSI model, and thus, allowed for model comparisons using the *χ*^*2*^ difference test [44] (Δ*χ*^*2*^). Because the quadratic slope intercept model was not nested, we also compared fit using information theoretic fit indices, which are suitable to comparing non-nested models (i.e., Akaike Information Criterion (AIC) and Bayesian Information Criterion (BIC), with lower values indicating a better fit [45]. Models were estimated using the full information maximum likelihood estimator in Mplus 7.1 [46]. Overall model fit was evaluated by examining multiple indices using conventional guidelines for evaluating good model fit: non-significant *χ*^*2*^ likelihood ratio test; Comparative Fit Index (CFI) and Tucker-Lewis Index (TLI) ≥ .95; Root Mean Square Error of Approximation (RMSEA) < .05 [47, 48].

After determining the most appropriate statistical model to capture BPD symptom development, we regressed the intercept and slope latent variables on temperament and demographic covariates of minority race and family poverty at Wave 1. The goal of this procedure is to determine how temperament impacts individual differences in the starting point of BPD growth trajectories (the intercept), and their pattern of development over time (the slope), net of background demographics. To evaluate the utility of including multiple informants of child temperament in predicting BPD symptom development, we examined the magnitude and significance of the regression coefficients for both parent- and teacher-reports of temperament, when both are included in the model simultaneously.

## Results

### Comparing growth models of BPD symptom development

Table 1 provides descriptive statistics and correlations among all study variables. As can be seen in Table 1, there is evidence for both continuity and change in BPD symptoms in our sample (as indicated by modest correlations among BPD symptoms measured at different waves), as well as evidence for small but meaningful correlations between temperament (particularly emotionality) and later BPD symptoms.

**Table 1:** **Descriptive statistics and bivariate correlations for all study variables**

	1	2	3	4	5	6	7	8	9	10	11	12	13	14	15	16
**Demographic covariates**																
1. Minority race	1															
2. Family poverty, wave 1	0.35	1														
**Temperament, parent-report at wave 1**																
3. Emotionality	0.04	0.08	1													
4. Activity	-0.07	-0.04	-0.11	1												
5. Sociability	0.10	0.08	0.26	-0.24	1											
6. Shyness	0.08	0.09	0.26	-0.16	0.31	1										
**Temperament, teacher-report at wave 1**																
7. Emotionality	0.12	0.07	0.12	0.01	0.06	0.004	1									
8. Activity	-0.01	-0.04	-0.01	0.17	-0.09	-0.17	0.08	1								
9. Sociability	0.04	0.08	0.04	-0.09	0.15	0.09	0.33	-0.38	1							
10. Shyness	-0.02	0.03	0.01	-0.12	0.11	0.27	0.17	-0.42	0.51	1						
**BPD symptoms, child/youth report**																
11. BPD symptoms, Age 14	0.09	0.06	0.13	0.02	0.06	-0.01	0.11	0.05	0.04	-0.04	1					
12. BPD symptoms, Age 15	0.12	0.09	0.12	0.04	0.04	-0.03	0.11	0.01	0.05	-0.04	0.54	1				
13. BPD symptoms, Age 16	0.13	0.09	0.11	0.04	0.09	-0.03	0.15	0.04	0.05	-0.06	0.49	0.58	1			
14. BPD symptoms, Age 17	0.13	0.08	0.11	0.04	0.06	-0.01	0.12	-0.02	0.07	-0.01	0.44	0.52	0.59	1		
15. BPD symptoms, Age 18	0.16	0.10	0.07	0.04	0.08	-0.02	0.10	-0.03	0.03	-0.02	0.38	0.42	0.49	0.56	1	
16. BPD symptoms, Age 19	0.09	0.10	0.08	0.04	0.05	-0.04	0.12	-0.004	0.02	0.02	0.39	0.40	0.42	0.50	0.54	1
Mean/proportion (%)	58.8%	38.9%	12.93	18.81	1.83	4.83	8.80	14.30	1.82	4.32	2.44	2.49	2.36	2.17	2.00	1.89
SD	−	−	4.91	3.76	1.11	2.20	4.52	3.99	1.11	2.17	1.90	1.90	1.89	1.86	1.79	1.67

*Note.* BPD = borderline personality disorder. *p* < .05 when *r* ≥ |0.05|.

**Figure 1:**
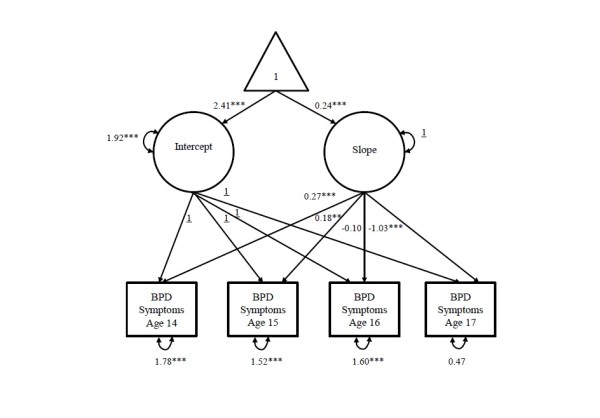
**Free curve slope intercept model.** This figure illustrates the unstandardized parameter estimates for the orthogonal free curve slope intercept model of borderline personality disorder symptom development from age 14 to age 19. Circles indicate latent intercept and slope variables and squares indicate manifest (observed) variables. The triangle represents the intercept factor, and the loadings emanating from this variable are the intercept and slope means. Double-headed arrows (slings) represent variances and single-headed (straight) arrows are regression paths. Underlined values indicate parameter was fixed to one. The covariance between the latent intercept and slope variables is not shown because this parameter was fixed to zero. All estimated parameters are significant at *p* < .001 with the exception of the slope loading for BPD symptoms at age 17, which was nonsignificant (0.09, *p* > .05).

Table 2 presents model fit information for the seven growth models. As can be seen in Table 2, the FCSI model provided an acceptable fit to the data. Additionally, as demonstrated by the statistically significant χ^2^ difference tests displayed in Table 2, the FCSI model provided a significantly better fit to the data compared to the FM, FM-Shift, linear slope intercept model, and MANOVA models. The quadratic slope intercept model appeared to provide good fit to the data and had a lower AIC and BIC compared to the FCSI model (AICs = 41255.27 vs. 41249.32; BICs = 41341.26 vs. 41341.04 for the FCSI and quadratic models, respectively). However, the estimated variance associated with the intercept latent variable was negative indicating an improper solution. It should also be noted that the FCSI model captures non-linear (e.g., quadratic) growth with fewer estimated parameters compared to the quadratic slope intercept model and is, therefore, more parsimonious. The FCSI model was retained and Figure 1 displays the unstandardized parameter estimates for this model.

**Table 2:** **Fit statistics for growth models**

	χ ^2^	df	TLI	CFI	RMSEA	RMSEA (90% CI)	AIC	BIC	Δχ ^2^	df, Δχ ^2^
Free curve slope intercept model	78.78***	12	0.98	0.98	0.05	0.04-0.06	41255.27	41341.26		
Factor model with means	223.96***	14	0.94	0.95	0.08	0.07-0.09	41396.44	41470.97	145.18***	2
Factor model with means-shift	221.85***	13	0.94	0.95	0.08	0.07-0.09	41396.33	41476.59	143.07***	1
MANOVA	237.05***	19	0.96	0.94	0.07	0.06-0.08	41399.54	41445.40	158.27***	7
MANOVA w/o compound symmetry	258.14***	14	0.93	0.94	0.09	0.08-0.10	41430.63	41505.16	179.36***	2
Linear slope intercept model	150.17***	16	0.97	0.97	0.06	0.05-0.07	41318.66	41381.72	71.39***	4
Quadratic slope intercept model^*Ep*^	70.83***	11	0.98	0.98	0.05	0.04-0.06	41249.32	41341.04	--	--

*Note.* The Free Curve Slope Intercept Model was the base model against which the other forms of growth were compared using χ^2^ difference tests (Δχ^2^). The quadratic slope intercept model was not nested within the FCSI model so the χ^2^ difference test was not conducted. ^*Ep*^Estimation problems occurred when estimating the quadratic slope intercept model: the intercept latent variable had a negative error variance. ***p < .001.

**Table 3:** **Prospective associations between temperament and growth factors of BPD symptoms across ages 14 through 17**

	BPD symptom growth factors			
	Intercept		Slope	
Predictors	*Β*	*t*	*β*	*t*
**Demographic covariates**				
Minority race	0.16	5.38***	-0.04	-0.77
Family poverty, wave 1	0.07	2.30*	-0.01	-0.29
**Temperament, parent-report at wave 1**				
Emotionality	0.12	4.01***	0.08	1.74
Activity	0.06	2.26*	-0.01	-0.27
Sociability^*†*^	0.07	2.37*	-0.04	-0.76
Shyness	-0.11	-3.45***	0.04	0.75
**Temperament, teacher-report at wave 1**				
Emotionality	0.11	3.77***	0.02	0.45
Activity	-0.06	-1.70	0.11	2.26*
Sociability^*†*^	0.02	0.61	0.12	2.25*
Shyness	-0.04	-1.24	-0.12	-2.18*
**Total R** ^**2**^	0.09	5.81***	0.04	2.21*

*Note.* ^*†*^Sociability was scored such that higher values indicate lower levels. *p < .05; **p < .01; ***p < .001.

The unstandardized parameter estimates for the intercept variance and slope loadings can be examined to determine the relative amounts of trait and trajectory components in BPD symptoms across adolescence. The intercept variance estimate (1.67, *p* < .001; expressed in units of the BPD scale, which, as noted earlier in the Methods section, has a range of 0 to 9) indicates significant within-individual differences in the elevation of BPD symptoms from age 14 to age 19, reflecting the trait component of the model. Since the intercept and slope factors are orthogonal (the covariance of between the intercept and slope variables was set to zero), the relative amount of variability due to the growth component of the model can also be examined. The amount of variability at each age due to the latent trajectory was determined by examining the squared slope loadings: 0.56^2^ = 0.32 at age 14, 0.71^2^ = 0.50 at age 15, 0.45^2^ = 0.20 at age 16, 0.09^2^ = 0.01 at age 17, -0.33^2^ = 0.11 at age 18, and -0.44^2^ = 0.19 at age 19. Comparing the intercept variance and the squared slope loadings at each age, variability in BPD symptoms across all ages [ages 14 (0.32/1.67), 15(0.50/1.67), 16(0.20/1.67), 17(0.01/1.67), 18(0.11/1.67), and 19 (0.19/1.67)] reflects more inter-individual, trait differences and less intra-individual differences. Moreover, the residual variance at each assessment occasion is relatively stable (1.94, 1.43, 1.54, 1.58, 1.41, and 1.81 at ages 14 to 19, respectively), suggesting similar levels of measurement error across this period of adolescence.

### Temperament as a predictor of BPD symptom development

Table 3 presents the associations between parent and teacher reports of temperament and BPD symptoms across adolescence, controlling for minority race and public assistance. The final conditional model also demonstrated good fit to the data: χ^2^ (df = 52) = 86.60, *p* < .01; RMSEA = 0.02, 90% CI = 0.01, 0.03; CFI = .99 and TLI = .98; AIC = 29921.43 and BIC = 3011.41. The final model accounted for 9% of the variance (*t* = 5.81, *p* < .001) in the intercept factor of BPD symptoms and 4% of the variance (*t* = 2.21, *p* < .05) in the slope factor.

All parent reports of temperament domains were significantly associated with the intercept factor. Specifically, high emotionality, high activity, and low sociability at Wave 1 were significantly associated with higher elevations in BPD symptoms across ages 14 through 19. Parent reports of shyness at Wave 1 also predicted lower levels of BPD symptoms across this developmental window. Parent reports of temperament did not significantly predict the slope of BPD symptoms.

Additionally, all teacher reports of temperament significantly predicted the intercept or the slope factors, suggesting teacher reports also explain unique variance in adolescent BPD symptom development. Teacher report of emotionality at Wave 1 predicted unique variance in the intercept factor, indicating that emotionality as measured by teachers predicted elevated BPD symptoms across adolescence. Moreover, teacher reports of activity and sociability at Wave 1 predicted the slope factor, suggesting that high activity and low sociability predicted increasing BPD symptoms across ages 14 to 19. Teacher reports of shyness at Wave 1 negatively predicted the slope factor, indicating that teacher reports of shyness predicted a slower rate of growth across this age span.

**Figure 2:**
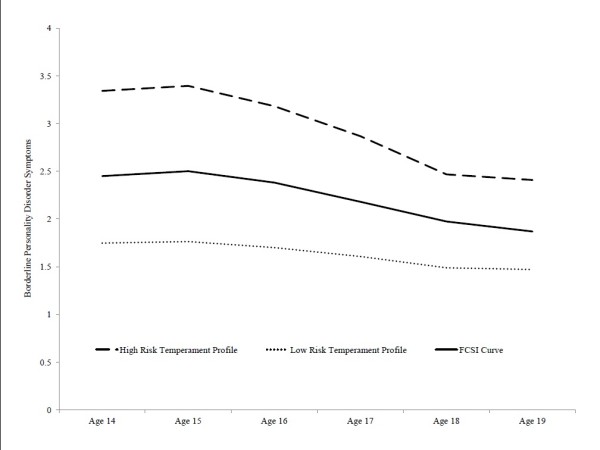
**Average curve for the orthogonal free curve slope intercept model (FCSI) and average curves for high and low risk temperament profiles.** The high and low risk profiles were created by adjusting the means for FCSI curve for girls/youth with scores in the 80th and 20th percentiles of parent- and teacher-reports of temperament.

Figure 2 depicts the effect of combining parent and teacher reports of child temperament in predicting the developmental trajectory of BPD symptoms. We created “high” and “low” risk developmental curves by adjusting the means for the average FCSI curve for girls/youth with scores in the 80th and 20th percentiles of parent and teacher reports of temperament. Specifically, the high-risk trajectory was adjusted by scores in the 80th percentile of parent and teacher emotionality, activity, and lack of sociability as well as scores in the 20th percentile of parent- and teacher-reported shyness (due to the inverse relationship between shyness and the intercept and slope factors). Conversely, the low risk trajectory was adjusted by scores in the 20th percentile of parent- and teacher-reported emotionality, activity, and lack of sociability as well as scores in the 80th percentile of parent- and teacher-reported shyness. As seen in Figure 2, temperamental risk has a notable impact on the development of BPD symptoms, presaging the development of symptoms of greater intensity throughout adolescence.

## Discussion

These analyses examined how childhood temperament impacts the developmental course of BPD symptoms throughout adolescence. We extensively evaluated various growth models of BPD symptoms across ages 14 through 19 and found that the FCSI model provided the best fit to the data. Comparing the assumptions of other growth models to those of the FCSI model extends our understanding of the course of BPD symptoms. Since the FM and FM-Shift models constrain the estimation of an intercept latent variable, the failure of these models suggests that there are significant amounts of inter-individual or trait-like differences in BPD symptoms from age 14 through age 19. Moreover, the failure of the MANOVA models to provide better model fit indicates that in addition to the within-individual trait component, a significant amount of within-person change in BPD symptoms also exists across this developmental period. Finding significant between- and within-person variability is consistent with our previous report of moderate levels of stability with some year-to-year fluctuations in BPD symptoms across ages 14 through 17 [14]. Lastly, the failure of the linear and quadratic slope intercept models implies that a chronometric rate of change does not adequately capture the within-person change observed in the development of BPD symptoms across adolescence.

Our finding that the FCSI model best captures the form of adolescent BPD symptom development is consistent with Wood’s (2011) re-analysis of temperament data measured in the first 5 years of life. Taken together, these findings support the notion that temperament and personality reflect processes characterized by “multiple short-term bursts of change, plateaus, or asymptotes in performance” across development [20]. Findings from the FCSI model reveal that the typical course of adolescent BPD symptoms reflects: (1) a principal component of trait-like stability (e.g., children with elevated symptoms at age 14 continue to have elevated symptoms at ages 15, 16, and 17 compared to children with lower symptoms) and (2) a relatively minor component of state-like changes from year to year (e.g., a pattern of increasing/decreasing symptoms within an individual child across time). On average, BPD symptoms appear to increase through age 15 and then decline through age 18 with a slight leveling off from age 18 to 19. The pattern of decline through late adolescence has been demonstrated in other community samples [10–12, 14] and likely reflects the normative decline in negative affectivity and disinhibition that is observed during this developmental period [15, 16]. The current study extended previous findings by critically evaluating the fit of various statistical models providing an enhanced characterization of the form of BPD symptom development across adolescence.

We also found support for our hypothesis that childhood temperament and adolescent BPD symptoms likely reflect the same basic underlying dimensions. Temperament predicted higher elevations of BPD symptoms as well as increases in symptoms across time. Emotionality was the strongest predictor of elevated BPD symptoms, with both parent- and teacher-reports predicting this pattern of development across time. Additionally, parent-reports of high activity, low sociability, and low shyness predicted elevated levels of symptoms while teacher reports of these dimensions predicted increases in the rates of symptoms across time. These findings are consistent with previous work documenting the links between infant temperament dimensions of emotionality and activity predicting BPD symptoms 28 years later [7]. Finding that low sociability and low shyness predicted the developmental course of BPD symptoms may suggest that these temperament dimensions reflect, at least in part, aspects of antagonistic and disinhibited personality traits. However, it is important to note that our findings do not suggest a unique relationship between this child temperament profile and adolescent BPD symptoms and it is likely that these temperamental dimensions increases risk for disorders across both the internalizing and externalizing spectra. Consequently, early intervention efforts targeting such broadband risk factors could prevent a host of internalizing and externalizing factors. Future work is needed to explicate the specific mechanisms leading to a particular adverse outcome that will also lead to more tailored intervention and prevention strategies.

Additionally, our results indicate significant value in gathering multiple informants when assessing child temperament. These findings demonstrate that both parent and teacher reports of temperament explain unique variance in the trajectory of BPD symptoms across adolescence. Parents and teachers have access to different, yet complementary information about the child’s temperament that is useful in predicting clinical symptoms assessed 5–12 years later.

This study is not without limitations. BPD symptoms were not measured prior to age 14 nor were temperament dimensions measured annually in adolescence so it is not possible to determine the co-occurring or reciprocal relationships among these constructs. However, the prospective nature of our assessments and the early age at which temperament was measured lessen concerns that girls’ BPD symptoms would be driving temperament. Another limitation concerns the generalizability of our findings. The sample only included girls so the findings may not generalize to male-only or mixed-gender samples. In addition, BPD was not measured repeatedly with a semi-structured interview but relied on interview guided self-reports. However, this measure has been widely used in adolescent community and psychiatric patient samples and its relationship to diagnosis is well established [38, 49]. Moreover, we measured the course of symptoms rather than the course of disorder. Given the nature of our community sample, few girls met the threshold required for diagnosis (5 out of 9 symptoms) in any given year. It is important to note, however, that this threshold is arbitrary and that the endorsement of even one BPD symptom is related to clinically significant distress and functional impairment [50]. It is useful to evaluate the impact of childhood temperament in this context. Specifically, when examining the impact of temperament on the developmental course of BPD, girls with high-risk temperament profiles had, on average, 1–2 more BPD symptoms when compared to girls with low-risk temperament profiles. Although the associations between temperament and BPD symptoms were significant, it is worth noting that childhood temperament accounted for a modest amount of variance in the development trajectory of BPD symptoms during adolescence. This low amount of variance may be due to: (1) the large span of time between the baseline assessment (occurring between ages 5–8) and the first assessment of BPD symptoms (age 14) as well as (2) the different informants providing information on temperament (parent and teacher reports) and BPD symptoms (child report).

Another limitation concerned the abbreviated Sociability assessment in our sample. Specifically, Sociability was measured with only one item in parent and teacher reports, which may have reduced the validity of this index. However, we do have evidence supporting the validity of this index. For instance, the pattern of bivariate correlations between parent and teacher reports of temperament dimensions demonstrated construct validity for the Sociability index (Table 1). Parent and teacher reports of Sociability are more strongly correlated (*r* = 0.15, *p* < .05) than parent-reported Sociability and any other teacher-reported temperament dimension, reflecting both convergent and discriminant validity, respectively. Moreover, the magnitude of this association is within the range obtained for the other temperament dimensions we assessed (i.e., *r*’s = 0.12, 0.17, and 0.27 for Emotionality, Activity, and Shyness, respectively). Including Sociability was also of theoretical significance as it allowed us to examine associations between the full complement of temperament dimensions as articulated by Buss and Plomin (1984) and BPD symptom development. Removing this scale would only offer a fragmented approach to examining these relationships. Importantly, this brief measure of Sociability did indeed predict BPD symptom development, which supports the utility of this index in our sample.

Despite these limitations, our study has several important strengths. The validity of our findings is supported by our prospective design, such that we assessed temperament prior to our measurement of borderline personality disorder symptoms. Additionally, we utilized a multiple informant design, and including parent and teacher ratings of temperament and child reports of BPD symptoms. This design feature eliminates concerns about shared method variance accounting for the observed pattern of associations. Finally, the use of community participants rather than clinical patients ensures that the prospective associations observed are more representative of the development of BPD that unfolds in the general population and are less likely to be biased by the effects of treatment or characteristics that are specific to those who seek treatment.

## Conclusions

In sum, BPD symptoms in adolescence reflect trait-like differences between youth with less within-person variability across time. Symptoms appeared to peak by age 15, decline through age 18, and remain steady between ages 18 and 19. Childhood temperament dimensions of emotionality, activity, low sociability, and shyness predict adolescent BPD symptom development. Specifically, children with high levels of emotionality may be at risk for particularly elevated course of BPD symptoms across adolescence. Parent and teacher informants provide unique information about the course of BPD symptoms, suggesting the utility of collecting child assessments using multiple informants.
